# Evolution and ontogeny of bacteriocytes in insects

**DOI:** 10.3389/fphys.2022.1034066

**Published:** 2022-11-25

**Authors:** Mauricio E. Alarcón, Priscila G. Polo, Sevim Nur Akyüz, Ab. Matteen Rafiqi

**Affiliations:** Department of Molecular Biology, Beykoz Institute of Life Sciences and Biotechnology, Bezmialem Vakif University, Istanbul, Turkey

**Keywords:** endosymbiosis, bacteriocytes, insects, evolution, development

## Abstract

The ontogenetic origins of the bacteriocytes, which are cells that harbour bacterial intracellular endosymbionts in multicellular animals, are unknown. During embryonic development, a series of morphological and transcriptional changes determine the fate of distinct cell types. The ontogeny of bacteriocytes is intimately linked with the evolutionary transition of endosymbionts from an extracellular to an intracellular environment, which in turn is linked to the diet of the host insect. Here we review the evolution and development of bacteriocytes in insects. We first classify the endosymbiotic occupants of bacteriocytes, highlighting the complex challenges they pose to the host. Then, we recall the historical account of the discovery of bacteriocytes. We then summarize the molecular interactions between the endosymbiont and the host. In addition, we illustrate the genetic contexts in which the bacteriocytes develop, with examples of the genetic changes in the hosts and endosymbionts, during specific endosymbiotic associations. We finally address the evolutionary origin as well as the putative ontogenetic or developmental source of bacteriocytes in insects.

## Introduction

Microorganisms interact actively with higher living forms and have profoundly influenced the evolution of life on earth. For a microorganism, the entire body of a host presents multiple novel niches, where they thrive. The intestinal cavities of insects or the human gut are well-studied examples of this, where a vast array of bacteria, fungi, viruses, protozoa, and archaea live (Engel and Moran, 2013; Matijašić et al., 2020). When the microbial partner lives inside the body of the host, it is called an endosymbiont (Buchner, 1965). The endosymbiont can be either mutualistic, commensalistic, or parasitic; obligate or facultative; and intracellular or extracellular (Gupta and Nair, 2020). The higher organisms that host the endosymbiont are known to have a variety of suitable morphological adaptations to accommodate their “guests”; these include fermentation chambers, buccal pockets, blind sacs, crypts in their gut, diverticula, light organs, mycetocytes, mycetomes, bacteriocytes, bacteriomes, root nodules, stem nodules, cuticular organs, and trophosomes (Koch, 1967; Janke et al., 2022). The broader occurrence among animals in general, and comparative morphology of the symbiotic organs has been reviewed elsewhere (Douglas, 2020). The terms “bacteriocyte” or “mycetocyte” are used to describe a cell of host origin that houses intracellular endosymbionts (Buchner, 1965). Bacterio-is a prefix meaning bacteria, myceto-is a term used for fungi, and -cyte is used for cells. Therefore, bacteriocytes and mycetocytes are specialized cells containing endosymbiotic bacteria or fungi. These cells at times are aggregated forming organs known as bacteriomes or mycetomes. How these cells or structures evolved and whether the endosymbionts facilitated their evolution remains unknown.

Endosymbionts of the present day, living intracellularly, had an existence outside the cell at an earlier stage of their biological evolution (Sagan, 1967; Smith, 1979). The theory of “symbiogenesis” has beautifully envisioned how this may have occurred (Merezhkowsky, 1909; Sagan, 1967). However, the details of the mechanism at the molecular level remain elusive. For establishing intracellular symbiosis, first, microorganisms and a potential host must share some ecological context to have the chance to meet and engage in a relationship with each other. Subsequently, the endosymbiont and the host undergo adaptations at the morphological, cellular, immune, genetic, and genomic levels (Pais et al., 2008; Brinza et al., 2009; Douglas et al., 2011; Weiss et al., 2012; Kupper et al., 2016; Luan et al., 2016; Li et al., 2017; Miravet-Verde et al., 2017; Sabater-Muñoz and Toft, 2020; Perreau and Moran, 2021). Together, these adaptations help the endosymbiont in the transition from an extracellular free-living environment to one that is intracellular. Once inside the host cell, the interacting partners continue to undergo changes. Endosymbionts must evolve novel traits to evade defense mechanisms of the host and often lose the necessity for some features essential for a free-living state (Toft and Fares, 2008; Gilbert et al., 2012). Common outcomes of these are “directional selection” for immune responsiveness and “relaxed selection” for non-essential features (Kinjo et al., 2021). These processes lead to the reduction in the genomes of the endosymbiont, horizontal gene transfer from endosymbionts to host, or changes in the individual gene regulatory networks of the partners (Braendle et al., 2003; Nikoh and Nakabachi, 2009; McCutcheon and Moran, 2012; Matsuura et al., 2015; Rafiqi et al., 2020). Genetic and epigenetic changes at the individual gene expression and function levels have been reported for genes that are directly involved in endosymbiont maintenance, transmission, bacteriocyte development, and germline formation (Matsuura et al., 2015; Rafiqi et al., 2020). At the same time, genes of the host are influenced by the presence of endosymbiont affecting processes that maintain their existence in the host (Schwemmler, 1974; Rafiqi et al., 2020). Therefore, changes occur at multiple levels that eventually help the endosymbiont integrate into the biology of its host (Rafiqi et al., 2022).

Significant knowledge has been accumulated over the last century through studying endosymbiosis in insects (Blochmann, 1882; Tannreuther, 1907; Šulc, 1910; Shinji, 1919; Lilienstern, 1932; Koch, 1967; Schwemmler, 1974; Ishikawa, 1985; Lambiase et al., 1997; Moran and Telang, 1998; Sauer et al., 2000; Fukatsu and Hosokawa, 2002; Braendle et al., 2003; Estes et al., 2009; Wernegreen et al., 2009; Moran and Jarvik, 2010; Koga et al., 2012; Shigenobu and Stern, 2013; Vigneron et al., 2014; Hosokawa et al., 2016). Insects are easy to rear, handle and present least ethical considerations, which has made them a very useful model taxon for research. They are a diverse group and the largest class within animals with over one million described species (Grimaldi et al., 2005; Stork, 2018).

Bacterial endosymbionts are widely present across the class Insecta and are housed into bacteriocytes, localized in different parts of the body depending on the host (Buchner, 1965; Moran and Telang, 1998; Moya et al., 2008). The bacteriocytes are found mostly associated with the digestive tract in insects. For instance, in weevils (Coleoptera: Curculionidae) surrounding the foregut-midgut junction in larvae, mesenteric caeca in adults (Nardon, 1981; Lefevre et al., 2004) in psyllids (Hemiptera: Psyllidae) in the abdomen surrounding the gut (Fukatsu and Nikoh, 1998), in whiteflies in the gut (Hemiptera: Aleyrodidae) (Thao and Baumann, 2004), and ants (Hymenoptera: Formicidae) within the midgut (Sauer et al., 2000). Usually, intracellular endosymbionts live in the cytoplasm of the cells of the host. In rare cases, endosymbionts have found their way into eukaryotic subcellular compartments like the bacteria *Rickettsia* that has been found in the nuclei of cells of some species of Hemiptera (planthoppers from Cixiidae family), Psocoptera (Liposcelididae), and Isoptera (Kalotermitidae, Rhinotermitidae) (Grandi et al., 1997; Perotti et al., 2006; Arneodo et al., 2008), and the mitochondria of a mirid species (Heteroptera: Miridae) (Chang and Musgrave, 1970). Overall, research into insects has contributed greatly to the understanding of endosymbiosis, leading to important insights into the origin, physiological processes, ecological interactions, genetic interactions, evolution, and developmental integration of endosymbionts (Buchner, 1965; Klepzig et al., 2009; Rafiqi et al., 2022).

Various researchers have assumed the putative ontogenetic origins of the bacteriocytes in multicellular animals, which include specialized adipocytes (Glaser, 1920), mesoderm (Katz et al., 2011), oenocytes (Lambiase et al., 1997), neoblasts (Dirks et al., 2012), archaeocytes (Simpson, 2012), and gut epithelium (Thorington et al., 1979). During embryonic development, a series of morphological and molecular changes define the fate of distinct cell types (Gilbert and Barresi, 2017). The ontogeny of bacteriocytes is intimately linked with the evolutionary transition of endosymbionts from extracellular to intracellular environment, for which several hypotheses have been forwarded (Flórez et al., 2015; Gray, 2017). To fully understand the ontogeny of bacteriocytes and the evolutionary transition to intracellularity, several questions need to be answered. For example, how do the endosymbionts avoid immune response and apoptosis of the host? How do they overcome physical barriers and outcompete other symbionts? and most importantly, how do they reach areas in the body of the host that allow them to transmit from generation to generation? Understanding the ontogeny of bacteriocytes thus requires multidisciplinary approaches.

Global changes at the genomic level, such as genome reduction in endosymbionts, horizontal gene transfer from bacteria to the eukaryotic hosts, immunomodulation, nutritional interdependence, and regulation of endosymbiont titer have been thoroughly reviewed elsewhere (Ishikawa, 1989; Wernegreen, 2002; Vogel and Moran, 2011; Gilbert et al., 2012; Eleftherianos et al., 2013; Skidmore and Hansen, 2017; López-Madrigal and Duarte, 2019; Perreau and Moran, 2021) and are beyond the scope of this review. Here, a special focus is given to the evolution of and the developmental mechanisms that specify bacteriocytes, i.e., cells that harbor bacterial intracellular endosymbionts in insects. In the sections that follow, we first classify the endosymbiotic occupants of bacteriocytes highlighting the complex challenges they pose to the host. Then, we recall the historical account of the discovery of bacteriocytes. We then summarize the molecular interactions between the endosymbiont and host. In addition, we illustrate the genetic contexts in which the bacteriocytes develop with examples of the genetic changes in the hosts and endosymbionts during specific endosymbiotic associations. We finally address the evolutionary origin as well as the putative ontogenetic or developmental source of bacteriocytes in insects.

## The endosymbiotic occupants of the host cells

Within host cells, endosymbionts have a complex relationship with the host, which goes beyond their classification as obligate or facultative (Ishikawa, 1989). Obligate endosymbionts are essential for the host’s survival, often live and replicate exclusively inside of the host cells, and are maternally transmitted. Facultative endosymbionts are not required for host survival, can replicate inside or outside the host cells, can be acquired from the environment, and many can be cultured outside the host (Koch, 1967). In contrast, the obligate endosymbionts’ particular lifestyle makes it difficult to culture them outside the host in most cases (Masson and Lemaitre, 2020). Additionally, endosymbionts are known to switch between different lifestyles. For instance, the facultative *Sodalis* (Enterobacteriales: Enterobacteriaceae) in the tsetse fly can be found inter- and intracellularly in different types of tissues of the fly (Balmand et al., 2013). Similarly, *Spiroplasma* symbionts associate intracellularly as well as extracellularly (Regassa and Gasparich, 2006). *Serratia symbiotica* endosymbiont of the aphid *Periphyllus* is found in bacteriocytes but becomes extracellular by massively infecting the digestive tract and other tissues during embryogenesis (Renoz et al., 2022). This suggests that endosymbionts can exhibit evasive behaviour and escape strict compartmentalization (Renoz et al., 2022). In addition, some endosymbionts may manipulate host sexuality and reproduction, altering the fitness of their host (Himler et al., 2011; Kageyama et al., 2012; Gil and Latorre, 2019). For example, *Wolbachia, Cardinium*, and *Rickettsia* affect the reproductive properties of their insect hosts by inducing parthenogenesis, male-killing, feminization, and cytoplasmic incompatibility (Kageyama et al., 2012). When a male infected with *Wolbachia* mates with an uninfected female, the eggs are sterile (Werren et al., 2008; Giorgini et al., 2009; Saridaki and Bourtzis, 2010). Likewise, some species of *Spiroplasma* bacteria kill male offspring during embryogenesis which causes a bias in female sex ratio of their insects’ host as observed in *Drosophila* flies, ladybird beetles, and butterflies (Jiggins et al., 2000; Tinsley and Majerus, 2006; Bentley et al., 2007).

During the course of adaptation to novel niches, the endosymbiont may be newly acquired, lost, or replaced (Bennett and Moran, 2015; Sudakaran et al., 2017). In the Philaenini tribe of spittlebugs (Auchenorrhyncha: Cercopoidea) *Zinderia*, an endosymbiont with a degenerate genome, has been replaced by a novel symbiont closely related to *Sodalis glossinidius* (Enterobacteriaceae) (Koga et al., 2013). This replacement is accompanied by the co-evolution of distinct type of bacteriocytes (Koga et al., 2013). In Cerataphidini aphids in contrast to most of their relatives, *Buchnera aphidicola* endosymbionts housed in bacteriocytes have been replaced by yeast-like extracellular symbionts (Fukatsu and Ishikawa, 1996). The replacement may also be triggered by physiological or ecological constraints, for instance, feeding habits of adults vs. immature stages as reported in *Hylobius abietis* and *H. transversovittatus* (Conord et al., 2008). In whiteflies two closely related species show distinct fates of bacteriocytes during embryogenesis; in *Bemisia tabaci* they are maternal whereas in *Trialeurodes vaporariorum* embryonic cells perform the bacteriocyte function (Xu et al., 2020). The host, on the other hand, often regulates the population of the endosymbiont, varying their numbers depending upon the context. The context can be a different developmental stage, morph, sex, genotype, the diet of the host, and temperature as well as the presence of other endosymbionts (Komaki and Ishikawa, 2000; Goto et al., 2006; Bermingham et al., 2009; Chen et al., 2009; Vogel and Moran, 2011; Koga et al., 2012; Zhang et al., 2016; Li et al., 2022). Overall, the endosymbionts exert different types of challenges to the host that must be overcome to sustain the endosymbiotic relationship. One of the main solutions to these, as we shall discuss in the next section, is compartmentalization of endosymbionts.

## The “room” to house endosymbionts

Robert Hooke who coined the term “cell” while observing the structure of cork must be credited with the first observation of a bacteriocyte in human lice in 1,664 (Hooke, 1665). Under his rudimentary microscopy, Hooke describes bacteriocytes as “liver of the lice” (Hooke, 1665). The structure was also described as a “lemon-yellow organ” by Swammerdam in 1,669 who termed it the “stomach gland” (Swammerdam, 1737). Almost 200 years later, a few researchers, most prominently Leydig and Huxley observed cells with “granular protoplasm” in the body cavity of aphids which they designated as “pseudo vitellus” (Leydig, 1850; Huxley, 1858). In the following years, several researchers described the presence of these cells in hemipterans often calling them a “non-living substance” (Balbiani, 1866; Mecznikow, 1866; Witlaczil, 1884; Will, 1888; Tannreuther, 1907). However, it was Blochmann who suggested that the “peculiar cells” house bacteria, while observing *Camponotus ligniperda*, *Blattella germanica*, and *Blatta orientalis,* (Blochmann, 1882; Blochmann, 1887). In 1910 the zoologist Pierantoni and the embryologist Šulc independently interpreted the “pseudo vitellus” of Huxley to be a primitive gland organ in the gut tube populated with intracellular symbiotic microbes which they called “yeast fungi” (Pierantoni, 1910; Šulc, 1910). Šulc also introduced the term “mycetome” meaning “fungus-organ” because he thought the microbes inside these cells were yeast (Šulc, 1910). Paul Buchner, referred to as the founder of systematic symbiosis research, found that the bacteria harboured in specialized cells (i.e., bacteriocytes) assist the insect in food digestion (Buchner, 1965).

Bacteriocytes are found in species of several orders of insects such as Blattodea, Coleoptera, Diptera, Hemiptera, Hymenoptera, and Psocodea (Cornwallis et al., 2022; Nozaki and Shigenobu, 2022). Outside of the class Insecta, bacteriocyte-like cells are found scattered throughout the phylogeny of multicellular organisms (Douglas, 2020). Examples of this include giant multinucleate hypertrophied feeding cells present in infected plants destined to house the parasitic root-knot nematodes *Meloidogyne* (Caillaud et al., 2008), large ovoid translucent cells lacking either cytoplasm or organelles with *Gammaproteobacteria* in marine worms of the genus *Osedax* (Katz et al., 2011), large cortical polyploid cells, which house the arbuscular mycorrhizal fungus *Gigaspora margarita* in the roots of the clover *Medicago truncatula* (Carotenuto et al., 2019), or the cells of legume nodules which are grotesquely enlarged to accommodate *Rhizobium* endosymbionts (Maróti and Kondorosi, 2014). The presence of these type of cells in distinct eukaryotic lineages implies their multiple origins similar to that in insects (Moya et al., 2008).

Bacteriocytes are relatively large cells (approximately 100 μm in diameter) with a large nucleus (20 μm in diameter) (Koch, 1967; Douglas, 1998), and are highly polyploid (Braendle et al., 2003; Nakabachi and Moran, 2022; Nozaki and Shigenobu, 2022). The cell volumes of bacteriocytes change depending on their ploidy levels (Orr-Weaver, 2015). In the rare case of the brown planthopper, *Nilaparvata lugens* (Hemiptera: Delphacidae) fungal endosymbionts are present in a single huge insect cell occupying 22% of the volume of the host abdomen that possesses a thick cell wall (Wang et al., 2021). Whereas at the same time, the bacteriocytes can differ in their ploidy level based on their strategy of reproduction and sex. For example, viviparous female aphids have higher ploidy levels than oviparous females, and males have overall lower ploidy levels than females (Humphreys and Douglas, 1997; Simonet et al., 2016; Nozaki and Shigenobu, 2022). In the pea aphid *Acyrthosiphon pisum* (Hemiptera: Aphididae)*,* housing the symbiont *B. aphidicola*, bacteriocytes have larger than normal nucleoli, which appear to enlarge throughout the lifecycle in association with the rate of reproduction of the endosymbiont and polyploidy level (Nozaki and Shigenobu, 2022). Moreover, insect symbionts within the bacteriocyte are sometimes enclosed in a symbiosomal membrane (Duncan et al., 2014; Price et al., 2014; Filip et al., 2019). The bacteriocytes or mycetocytes appear in the phylogeny of life as cells that evolved a new function and therefore represent a novel cell fate. The emergence of this evolutionary novelty is thought to have revolutionized life on earth, especially during the diversification of higher life (Moya et al., 2008; Gibson and Hunter, 2010). In summary, bacteriocytes provide a safe compartment and mediate a range of intersecting inter-relations between the endosymbiont and their hosts.

### Molecular interactions between endosymbionts and hosts

Numerous molecular processes are affected by the association between the endosymbiotic partners. Examples of these are amino-acid complementation, methylation, ammonia metabolism, chaperones and protein folding regulation, immune regulation, cell adhesion, cell transport, cell-cycle, apoptosis, and germ cell regulation. Amino acid complementation between endosymbionts and hosts has been the hallmark of endosymbiosis highlighted by many years of research (Hansen and Moran, 2014; Feng et al., 2019; Pers and Hansen, 2021). The complementation of amino acid biosynthesis pathways between the genomes of the aphid host, the cellular endosymbiont *Buchnera*, and the gut symbionts are often concomitant with the phytobiome of the plant that the host insect feeds upon (reviewed in Hansen and Moran, 2014). In addition, methylation mediated by endosymbiont has been shown as a regulator of the expression of key host genes in bacteriocytes, which affect aphid hosts’ selection of plants for feeding (Kim et al., 2018). Some specific examples of amino acid complementation include i) The aphid host which may play a role in biosynthesizing and thus regulating ornithine for *Buchnera*, whereas many *Buchnera* taxa have lost the genes responsible for *ornithine* biosynthesis (Hansen and Moran, 2014), ii) The case of *B. tabaci* populations from Asia, where several endosymbiont genomes sampled have lost major components of the *vitamin B* biosynthesis pathways, and presumably, the host supplements these to the endosymbiont (Zhu et al., 2022), iii) A recent study proving that the only metabolic function consistently retained in symbiont genomes of ants that have cellular endosymbionts, namely *Camponotus, Plagiolepis, Formica*, and *Cardiocondyla,* is the capacity to synthesise tyrosine implying that they supplement the host amino acid deficiency (Jackson et al., 2022).

Genes for ammonia metabolism are often up-regulated in the bacteriocytes; the *B. aphidicola* genome has limited capacity for the assimilation of ammonia (Shigenobu et al., 2000), and transcriptomic studies have shown that nitrogen derived from ammonia is primarily used in the host to produce amino acids (Hansen and Moran, 2011; Macdonald et al., 2012). Consistently, *A. pisum* genes up-regulated in bacteriocytes of insect hosts living on specialized host plants (Kim et al., 2018) complement *Buchnera* genes with glutamine synthetase, glutamate synthase, and the transporter ApGLNT1 used to recycle ammonia for amino acid biosynthesis (Hansen and Moran, 2011; Price et al., 2014).

Heat shock proteins and other chaperones appear frequently to be involved in establishing or maintaining bacteriocyte function; the white fly population native to Brazil contains endosymbiotic *Hamiltonella*, which have three mutations in the GroEL gene, a chaperone gene that regulates protein folding compared to *Hamiltonella* from invasive species insinuating the involvement of GroEL in this endosymbiotic association (Rossitto De Marchi et al., 2018). Temperature sensitivity of the endosymbiont density inside of each bacteriocyte in the case of aphids has been attributed to a point mutation in the endosymbiont genome that affects the ibpA gene, which encodes a universal small heat shock protein (Dunbar et al., 2007; Zhang et al., 2019). In oxidative stress situations, several chaperones known to be involved in protein refolding and maturation like clpB, dnaK, grpE, htpG, ibpA, and nfuA increase their expression in tsetse flies (Pontes et al., 2008).

Innate immunity of the host is also affected by bacteriocyte expression when a class of small anti-microbial peptides or AMPs a natural antibiotic began to be synthesized to resist symbiont invasion. The host can activate specific genes that produce these antimicrobial peptides, which appear to have evolved in plants as well as animal lineages that harbour endosymbionts (Shigenobu and Stern, 2013). These genes are differentially up-regulated in the bacteriocytes or bacteriomes (Shigenobu and Stern, 2013). In the plant *Medicago sativa*, antimicrobial peptides appear to be transported *via* the *bacA* transporter of the endosymbiont *Sinorhizobium meliloti* (Itoh et al., 2019). Although homologues of *bacA* are not reported from insect endosymbionts so far (Itoh et al., 2019), genes encoding small proteins, often cysteine-rich, start to be expressed during development in aphids, when *Buchnera* become engulfed in the bacteriocytes (Shigenobu and Stern, 2013). Similarly, in the bean bug, *Riptortus pedestris* specific sub-regions of the midgut have differentially up-regulated genes in the symbiotic individuals belonging to cysteine-rich secreted peptides in contrast to aposymbiotic individuals (Futahashi et al., 2013). In the weevil *Sitophilus,* an antimicrobial peptide selectively targets endosymbionts within the bacteriocytes and regulates their growth through the inhibition of cell division (Login et al., 2011).

In addition to AMPs, the bacteria in bacteriocytes avoid a variety of host immunological defense mechanisms, and in addition, bacterial populations are regulated as a result of the loss of infectivity-related genes specifically in these compartments (Ochman and Moran, 2001; Bennett et al., 2015; Perreau and Moran, 2021). In *Drosophila*, bacterial peptidoglycans bind to insect peptidoglycan receptors and activate two pathways concomitantly, one produces antimicrobial peptides to control the infection, and the conserved NF-κB immune signaling pathway, which produces peptidoglycan-destroying proteins (Zaidman-Rémy et al., 2006). An ortholog of the same peptidoglycan destroying gene in the weevil *S. zeamais* is up-regulated in the bacteriocytes and responds dynamically to the presence of a primary endosymbiont of this host (Anselme et al., 2006). Likewise, in the tsetse fly peptidoglycan recognition protein (pgrp)-lb responds to endosymbiont infection (Weiss et al., 2008). Artificial infection of *Buchnera* in *Drosophila* S2 cell cultures indicates that *Buchnera* is not able to survive the humoral immune system indicating that within *A. pisum* their compartmentalization in bacteriocytes is essential for escaping the host immune response (Douglas et al., 2011).

The host regulates genes for cell adhesion, storage, and transport; in the whiteflies, *B. tabaci* gene expression in bacteriocytes changes between the nymphal stage and the adult stage: cell adhesion genes are down-regulated while cell division, cell motility, and endocytosis/exocytosis genes are up-regulated in the adults compared to nymphs (Luan et al., 2016). A non-essential amino acid transporter 1, ApNEAAT1, of the *A. pisum* genome is capable of transporting proline, serine, alanine, and cysteine in an *in vivo* experimental setup implying its role in the transport of these amino acids from aphid hosts to *Buchnera* and vice versa (Feng et al., 2019). Bacteriocytes in the pea aphid show highly expressed genes for amino acid metabolism, transport, including genes for mitochondrial transporters and a gene encoding Rab, a G-protein that regulates vesicular transport; and genes for putative lysozymes that degrade bacterial cell walls (Nakabachi et al., 2005). *Burkholderia insecticola* gut symbiont of the insect *Riptortus pedestris* (Hemiptera: Alydidae) modulates host development and egg production by regulating the production of three hemolymph storage proteins and vitellogenin gene expression (Lee et al., 2017).

Bacteriocytes can be affected by the up-regulation of genes that control the cell cycle. Genes of the sugar metabolism are down-regulated when the system *A. pisum* - *Buchnera* is under nutritional deprivation, while homologues of the *putzig* gene are up-regulated in bacteriocytes. Such changes in gene expression influence the cell cycle, altering the size and number of bacteriocytes, and affecting the number of bacteria housed by bacteriocytes (Colella et al., 2018). In the case of white fly *B. tabaci*, the canonical arthropod telomere repeats TTAGG are increased in number in the bacteriocyte genome during development, and at the same time, telomere maintenance genes are over expressed (Luan et al., 2018), thereby enhancing the life expectancy of these cells of the host (Maciejowski and de Lange, 2017). Interestingly, the ancestral bacteriocytes may have been replaced by a different cell lineage in this case (Xu et al., 2020).

Interaction with the host autophagy and apoptosis pathways is essential for bacteriocyte function; in *B. tabaci* silencing the transcription factor Adf-1 (enriched in bacteriocytes) reduces the bacteriocyte number and activates their autophagy and apoptosis. This occurs more in males than females causing degeneration of the bacteriocyte in adult males (Wang et al., 2022). Vesicular trafficking pathway genes are overexpressed in bacteriocytes, such as Rab7 GTPase at symbiosomal membrane, which may be required for the degradation of *Buchnera* cells in older aphids (Simonet et al., 2018; Smith and Moran, 2020). In *Drosophila*, core autophagy proteins Atg1 and Atg8 and a selective autophagy-specific protein Ref (2) p negatively regulate *Wolbachia* in the hub, a male gonad somatic cell type, and *Wolbachia* effector protein, CifB, modulates autophagy (Xie and Klionsky, 2007). Intracellular bacteria undergo deubiquitination to escape recognition by host selective autophagy adapter proteins and survive in the cell (Deehan et al., 2021).

These examples indicate that the molecular interactions between the endosymbiont and host affect a wide range of metabolic and regulatory pathways of the partners. In addition, the expression of germline genes within the bacteriocytes has tempted some to speculate that the bacteriocytes may participate in guiding germ-cell migration (Lin et al., 2022). However, as we will show in the section on genes involved in bacteriocyte development, the interaction between the germline specification, and migration is intricately linked to the integration of the endosymbiont within the host.

### Regulation in extremely reduced genomes

One of the consequences of obligate endosymbiosis is reduction of genome size of the endosymbionts (Santos-Garcia et al., 2014; Miravet-Verde et al., 2017; Ankrah et al., 2018). This genomic reduction is accompanied by gene loss, which may have consequences and effects on transcriptional regulation (Miravet-Verde et al., 2017). In some cases of extreme genomic reduction, small RNAs may have taken over much regulatory function in the face of gene loss in the endosymbiont genomes; several endosymbiont genes that reportedly encode for small RNAs have been shown to regulate aphid genes (Thairu et al., 2018). Interestingly, there is post transcriptional regulation of coding genes as a possible mechanism since endosymbionts have lost many transcription factors and operons (Shigenobu et al., 2000; Ochman and Moran, 2001; Thairu et al., 2018). The sRNAs that are unique to specific *Buchnera* lineages may be important in the symbiont’s adaption to its specific aphid host lineage, whereas sRNAs conserved across taxa may be important for the general maintenance of the nutritional symbiosis, or general bacterial function (Thairu et al., 2018). Most of the ancestral transcription factors have been eroded in endosymbionts, most conserved operons have been fragmented and differential mRNA expression is not significant between life stages. Therefore it has been suggested that post-transcriptional processes may be the primary cause of differential gene regulation in endosymbionts, similar to what has been widely observed in mitochondria whereas differential protein expression maybe unlikely to be the regulatory mechanisms (Hansen and Moran, 2014). These data validate some computationally predicted regulatory elements and reveal a potential role for small RNAs in regulating gene expression in symbionts. The metabolic and regulatory interactions show how they remain interdependent but do not necessarily reveal the mechanisms for the establishment and integration of the endosymbiont in the host (Rafiqi et al., 2022). A thorough model for the molecular genetic basis of endosymbiosis requires understanding the specification and developmental origins of the bacteriocytes.

### Genes involved in bacteriocyte development

The relationship between the endosymbiont and host is a bidirectional association in which both the host and endosymbiont exert allowances and demands on each other (Smith and Moran, 2020). This affects processes at the phenotypic as well as at the genotypic level in both interacting partners. Most importantly, bacteriocyte development involves the interactions of multiple genes of the host and the endosymbiont; differences in the expression of these genes, or the timing of expression affects the bacteriocytes (Braendle et al., 2003). The genetic mechanisms of bacteriocyte specification, maintenance, and degeneration in multiple lineages are in the early stages of exploration.

Developmental integration of bacteriocytes has been proposed to be guided by highly conserved gene families (Rafiqi et al., 2022). In the lygaeid bug *Nysius plebeius* (Hemiptera: Lygaeidae), the gene *Ultrabithorax* (*Ubx*) is key in bacteriocyte differentiation, symbiont migration, infection, and localization in the abdominal segment (Matsuura et al., 2015). In the well-studied model of endosymbiosis between aphids and obligate bacterial symbiont *Buchnera*, *Ubx, Abdominal-A* (*Abd-A*), *Distal-less* (*Dll*), and *Engrailed* (*En*) are expressed in bacteriocytes from the blastoderm stage to the completion of embryonic development (Braendle et al., 2003). The expression of these genes is successive and follows the symbiont invasion of the syncytial nuclei at an early stage as well as the formation of bacteriocyte precursors at the posterior end of the blastoderm embryo (Braendle et al., 2003). The bacteriocyte differentiation occurs regardless of the presence of the endosymbiont and is suggestively controlled by gene expression exclusively from the aphid (Braendle et al., 2003). In the case of the carpenter ant *Camponotus floridanus* (Hymenoptera: Formicidae), bacteriocytes form in the posterior of the embryo close to cellularization of the blastoderm. Multiple Hox genes and germline genes are expressed in the bacteriocytes where *Ubx* is necessary for proper localization of the bacteriocytes, and *AbdA* is essential for their specification (Rafiqi et al., 2020). Moreover, germline genes, especially *oskar*, are thought to be involved in the segregation of the endosymbiont between the gut and the gonad, wherein a majority of the endosymbionts are destined to the gut and a smaller population is directed for transmission to the next generation (Rafiqi et al., 2020). Like aphids, the presence of endosymbionts in the case of ants does not seem to be necessary for bacteriocyte specification (Rafiqi et al., 2020). At the subcellular level changes in the bacteriocytes are possible, as observed in the cockroach *Blatella germanica* where profound structural changes appear in both the nucleous and cytoplasm of the bacteriocytes after the loss of bacteria (Grigolo et al., 1987). Therefore, highly conserved Hox genes are involved in bacteriocyte formation in species within at least two distinct orders of insects (Hemiptera and Hymenoptera). In the same species, endosymbionts also co-localize with germline genes to ensure transmission to the next generation. Both the Hox and germline specification pathways are well conserved across insects and therefore may have played a role in the evolution of bacteriocytes in multiple insect lineages.

### Evolutionary origin and ontogenetic source of the bacteriocytes

Association between hosts and their obligate endosymbionts presumably resulted from an ancient acquisition event from horizontally transmitted neutral or hostile microbes through a continuum leading to mutualism, followed by vertical transmission and co-evolution of the host and endosymbiont (Ewald, 1987; Dale et al., 2001; Drew et al., 2021). Comparative studies of microbiota have found relatedness between intracellular endosymbionts and “free-living” bacteria or extracellular symbionts (Wernegreen et al., 2009). There is evidence that free-living microbes have repeatedly been taken up as endosymbionts in multicellular eukaryotes (Hosokawa et al., 2016). Hypothetically, a potential host consumes the microorganisms from the environment and establishes a period of extracellular symbiosis in the gut. The transition from extracellular to intracellular then involves the formation of bacteriocytes most likely in the gut to “stabilize” this association. The “invaders” would lose their pernicious effects, and since they become serviceable to the host, the conflicts would gradually lead to mutual adaptation. However, the route by which the bacteria “infect” bacteriocytes may either be direct or may involve developmental steps during the formation of the gut cells. The intermediates between extracellular and cellular endosymbiosis point to possible mechanisms of transition between these states. In the case of the plataspid stink bug *Megacopta puctatissima*, endosymbionts are localised extracellularly in crypts formed in the lining of the midgut (Fukatsu and Hosokawa, 2002; Takeshita and Kikuchi, 2017). Although the crypts are extracellular, their function is similar to the bacteriocytes found inside the midgut epithelial cells in that they prevent endosymbiont purging during metamorphosis (Oishi et al., 2019). Similarly, the *gregarine* endosymbionts of the cat flea transit from extracellular to intracellular during metamorphosis to survive gut purging (Alarcón et al., 2011). In distantly related hydras (Phylum Cnidaria), endosymbiotic algae form clusters in the host endodermal cells inside large vacuoles, where some of them are digested by the host, and some are expelled and repeatedly re-engulfed leading to a process of continuous reinfection (Thorington et al., 1979; Rahat and Reich, 1984). Such repeated reinfection would in time select for an adapted symbiont and represent yet another intermediate state of transition to cellular endosymbiosis in the animal world. Although these examples fall short in explaining how intracellular endosymbionts evolved, they reveal possible scenarios in which a potential endosymbiont may come close to evolving an intracellular lifestyle (Thorington et al., 1979).

Several individual examples of the developmental origin of bacteriocytes have been elucidated at the embryonic level. In the cockroach *Periplaneta americana* (Blattodea), bacteriocytes are presumably derived from oenocytes or haemocytes, which are activated to perform phagocytosis (Lambiase et al., 1997). In the parthenogenetic aphid embryos, there are two types of cells that give rise to bacteriocytes. A first type is maternal wherein the nuclei of future bacteriocytes associate with bacteria from the follicular epithelial cells and form the bacteriocytes at a later stage (Braendle et al., 2003). A second population of bacteriocyte precursor cells starts from the posterior of the germband and migrates anteriorly to intercalate between the maternal bacteriocytes (Braendle et al., 2003). The bacteria enter into the bacteriocyte cytoplasm from the embryonic cytoplasm after the blastoderm stage and after the differentiation and polyploidization of the bacteriocytes, using the endocytic vesicle pathway (Szklarzewicz and Michalik, 2017). In *Balclutha calamagrostis* (Hemiptera: Cicadellidae), *Sodalis*-like bacteria are housed immediately proximal, outside the bacteriome in fat body cells (Kobiałka et al., 2018). Similarly, in *Fieberiella septentrionalis*, *Graphocraerus ventralis*, *Orientus ishidae*, and *Cicadula quadrinotata* (Hemiptera: Cicadellidae), *Sulcia* bacteria and fungal symbionts also reside in the fat body cells in addition to the bacteriome (Kobiałka et al., 2018). In some mealybugs of the subfamily *Pseudococcinae* the primary bacterial endosymbiont *Tremblaya princeps* housed within the bacteriocytes itself intracellularly contains a nested bacterial endosymbiont (Koga et al., 2012; von Dohlen et al., 2001).

In contrast to these cases of maternally defined bacteriocyte precursors, the pupal stage of the weevil *S. oryzae* intestinal stem cells under specific signals received during *Sodalis* infection triggers their differentiation into bacteriocytes (Maire et al., 2020). In the phylogenetically distant flatworm *Paracatenula galateia* (Platyhelminthes), bacteriocytes are constantly derived from aposymbiotic pluripotent neoblasts (Dirks et al., 2012). The host reproduces by asexual fragmentation and, vertically transmits numerous symbiont-containing bacteriocytes to its asexual progeny. The symbiont population is subjected therefore to reduced bottleneck effects. In the rare case of a vertebrate *Ambystoma maculatum,* the green algae *Oophila amblystomatis* endosymbionts invade multiple cell types in the host body, which could resemble proto-bacteriocytes in some aspects (Kerney et al., 2011).

In insects, after embryonic development, especially during the metamorphosis of larvae to pupae, significant changes are observed in the bacteriocytes. For example, in the *Camponotus* ants, the entire midgut transforms into a symbiotic organ (Hammer and Moran, 2019). The white fly, *B. tabaci*, inherits bacteriocytes maternally, consequently, multiple factors alter cellular and molecular processes that regulate bacteriocyte formation. Bacteriocytes are dramatically remodeled during the developmental transition from nymph to adulthood (Luan et al., 2016). Whereas in adult females cell division and proliferation of bacteriocytes occurs, in the bacteriocytes of adult males autophagy and apoptosis are induced (Li et al., 2022). In the females, there is also some disaggregation at this time possibly because each oocyte needs to obtain at least one bacteriocyte (Szklarzewicz and Moskal, 2001; Coombs et al., 2007; Luan et al., 2016). After a certain stage, the bacteriocytes can undergo apoptosis or autophagy in some lineages. This process enables the reuse of molecules of the host and helps conserve energy (Vigneron et al., 2014; Wang et al., 2022). These cells can be recycled to form an ordinary digestive cell, which seems to occur by autophagy of the endosymbiont (Gonçalves et al., 2020). In some cases, the degenerative features and remodeling of the bacteriocytes have been found to correlate with the process of metamorphosis in holometabolous insect hosts, enabling the maintenance of the bacteriocytes through adulthood (Maire et al., 2020). Exceptionally, the degeneration of bacteriocytes in aphids occurs by a distinct mechanism compared to other known examples. It starts with hypervaculation of the endoplasmic reticulum of the bacteriocyte, followed by a cascade of cellular stress responses involving potent lysosomal activity induced to create structures that engulf *Buchnera* (Colella et al., 2018). The developmental origin and fate of bacteriocytes are therefore complex, vary between lineages, and are not fully understood.

Since the endosymbionts mostly end up in the bacteriome or gut epithelia, how do they manage to transmit to the next generation? In some scale insects, planthoppers, heteropterans, hymenopterans, mallophagans, termites, and cockroaches the endosymbionts infect cystocytes in larval ovaries or oocytes in young adult female ovaries (Szklarzewicz and Michalik, 2017). However, in two cases known so far, the endosymbiont populations are segregated early during embryonic development. In the cereal weevils *Sitophilus oryzae, S. zeamais*, and *S. granarius*, endosymbionts are present in the posterior of the egg in close proximity to the germline and give rise to two distinct bacteriomes one that remains stable along with the germline, and another, which initially increases in bacterial load and is housed in the gut but eventually gets lost around 14th day after eclosion of the adults (Vigneron et al., 2014). In *C. floridanus*, endosymbionts are present in the posterior of the embryo and are enclosed in bacteriocytes that engulf them along with multiple zygotic nuclei (Rafiqi et al., 2020). Like in the weevils, the bacterial population is segregated into two at the time of cellularization of the syncytial stage embryos so that a large population is destined to the midgut inside the bacteriocytes, and a smaller population infects the germline (Rafiqi et al., 2020). Like *C. floridanus*, in the pea aphid, early blastoderm stage of sexual embryos, nuclei located at the posterior are enclosed by membranes together with the endosymbiont and give rise to the bacteriocytes (Braendle et al., 2003; Misof et al., 2014). However, the segregation of these has not been reported. Altogether, these data lead to the idea that there is a posterior region of the syncytium that is in close proximity to the future germline and is destined to the bacteriocytes, especially in the case of insects where bacteriocytes are specified during embryonic development.

## Discussion

Bacteriocytes’ phylogenetic distribution across the domains of life, distinct morphological features, and locations in the host indicate that different lineages do not have a discrete ancestral origin of these cells. It is advantageous to retain beneficial endosymbionts under conditions such as nutrient deficient diets. At the same time purging of gut contents during metamorphosis, can result in loss of beneficial microorganisms. Therefore, it is selectively advantageous for the host to have bacteriocytes, which provide a secure compartment for maintaining endosymbionts. At the developmental level, overall, the data so far indicate that bacteriocytes in insects are most often derived from cells originating at the posterior of the embryo. Some documented exceptions are hemipterans where fat body cells, oenocytes, and hemocytes, acquire bacteriocyte identity secondarily. There is also sufficient evidence indicating that bacteriocyte fate can be acquired as well as remodeled during metamorphosis. The cases where bacteriocytes are specified in the embryo from posterior cells provide the window of opportunity to investigate the origin of these cells in insects. We suspect that these cells may have at least in some lineages carried an ancestral developmental potential to repeatedly evolve into bacteriocytes. However, a thorough understanding of the biology of ancestral insects is fundamental for elucidating the evolutionary origin of bacteriocytes.

Insects are known to have evolved from pancrustaceans, which also include crustacean ancestors (Misof et al., 2014). Between the early Silurian and Devonian (444 Ma–359 Ma) pancrustaceans transited from marine to terrestrial environment, which coincided with the radiation as well as an increase in size and complexity of vascular plants (Engel and Grimaldi, 2004; Misof et al., 2014; Morris et al., 2018; Brookfield et al., 2021). Based on the morphology of the tracheal system of hexapods, it is generally accepted that insects evolved on land instead of the sea (Pritchard et al., 1993; Misof et al., 2014). At the origin of insects, the radiation of vascular plants offered a new source of food leading to the evolution of new modes of feeding to exploit different plant tissues (Hotton et al., 1996). During this time, organisms feeding on decaying plant matter, which contains cellulose-degrading bacteria, eventually would have paved the way for endosymbiotic associations that help with digesting plant material (Hotton et al., 1996), which in turn would have led to the emergence of bacteriocytes. In fact, fossil data indicate that sap-feeder hemipterans were well-represented close to the origin of insects (Shcherbakov, 2000; Li et al., 2017). Despite being specialist feeders, the herbivorous insects are surprisingly species-rich (Misof et al., 2014; Rainford and Mayhew, 2015; Kenneth and Jayashankar, 2020). This is understood to be in part because many of the representative sap-feeders have nutritional mutualistic dependence on bacterial endosymbionts, which may have played a role in their evolutionary success (Misof et al., 2014; Rainford and Mayhew, 2015; Cornwallis et al., 2022). Furthermore, in some lineages where bacteriocytes house the endosymbionts, an evolutionary switch to a predatory lifestyle correlates with a subsequent loss of bacteriocytes in their modern descendants (Ohbayashi et al., 2019; Weirauch et al., 2019). However, whether the emergence of bacteriocytes followed herbivory, or facilitated it, is not fully understood.

There are several competing ideas regarding the origin of herbivory in insects. One idea is that insects were herbivorous ancestrally and evolved predatory behaviour secondarily after losing access to nutritious plant material (Krimmel, 2011). However, more recent data has confirmed that insects were detritivorous and fungivorous first and later evolved both predatory as well as herbivorous behaviour (Rainford and Mayhew, 2015). Plant-feeding insects, due to the nutrient-deficient plant material, evolved endosymbiotic associations that provide cellulose-digesting enzymes and nutrients such as amino acids to the host (Douglas, 2009). Genomic and paleontological data indicate that herbivory is secondary within animals (Vermeij and Lindberg, 2000). Insects compared to animals in general carry bacteriocytes proportionately more often and may have evolved these along with intracellular endosymbionts secondarily within their lineage (Buchner, 1965). However, the presence or absence of bacteriocytes in the last common ancestor of insects cannot be proved merely by sampling for their presence. In case bacteriocytes were present in the last common ancestor of insects, they would have hosted endosymbionts and would have left cellular or molecular traces in their modern relatives. Indeed, lineages that have lost endosymbiosis still retain key genes or regulatory factors predisposing them to symbiosis in favorable conditions (Xu et al., 2020). It has been shown that intracellular endosymbionts are not always related to the gut microbes of that particular taxon (Wernegreen et al., 2009) and many endosymbionts evolved from environmental bacteria (Hosokawa et al., 2016). Intracellular endosymbionts may not be functionally replaced by the gut microbiota as shown in the cockroach *Blattella germanica* (Muñoz-Benavent et al., 2021). Within lygaeoid bugs, the families with gut symbiosis inside crypts are distinct from those families with bacteriocyte-associated endosymbiosis (Kuechler et al., 2012). Moreover, the crypt-based symbiosis may have been lost in the common ancestor of lineages where the bacteriocyte-associated systems secondarily evolved (Kuechler et al., 2019). These data, although limited, suggest that bacteriocytes are not merely an elaboration of gut microbiomes but evolved as a distinct novel feature within insects. In all cases, the ontogenetic origin of bacteriocytes in each lineage would hint at the ancestral states at the base of the insect tree.

**FIGURE 1:**
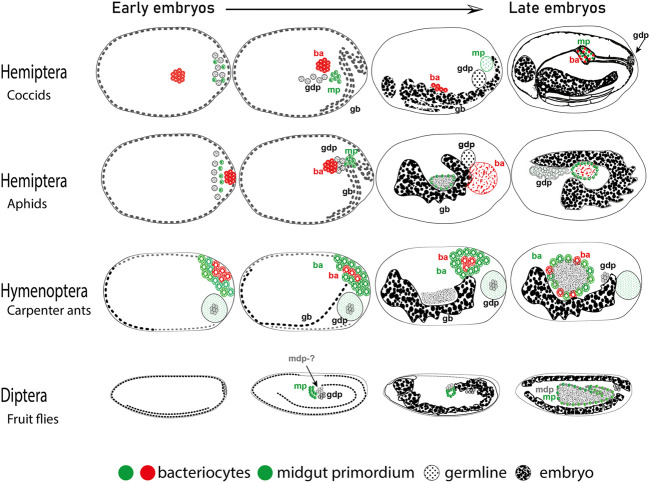
Cells destined to become bacteriocytes in embryos. Cartoons are based on (Shinji, 1919, Coccids, Lin et al., 2014, Aphids, Rafiqi et al., 2020, Carpenter ants, Campos-Ortega and Hartenstein, 2013- *Drosophila*). Depicted are embryonic stages from younger to older (left to right). Embryonic germband (gb), bacteriocytes (ba), germline destined pole cells (gdp), midgut destined pole cells (mdp), and midgut primordium (mp) are indicated. Anterior is to the left, dorsal is up.

As mentioned in the previous section, in the case of insects where bacteriocytes are specified during embryonic development, there is a posterior region of the syncytium that is destined to form the bacteriocytes. In multiple insect lineages, in close proximity to the germline precursors, there is a cell population between the germline proper and the midgut primordium that retains features of the germcells but is destined to the midgut epithelium (Shinji, 1919). Although disputed by some authors in earlier years (Schrader, 1922), it was substantiated by the work in *Drosophila* in the 1960s (Mahowald, 1962; Counce, 1963). At the same time in the coccids, weevils, sexual aphids, and ants, bacteriocytes are derived from the posterior of the embryo in an ontogenically similar fashion except that they are or become filled with endosymbiotic bacteria and are transported to the midgut (Figure 1) (Shinji, 1919; Heddi et al., 2005; Lin et al., 2014; Rafiqi et al., 2020). In lineages that contain endosymbionts, these cells become bacteriocytes whereas in other lineages they contribute to the midgut. Consistently, it has been shown that in the ant lineage within the genera *Formica* and *Cardiocondyla*, and in some lineages of beetles of subfamily Cassidinae*,* endosymbiosis has been repeatedly lost but the relics of the existence of bacteriocytes or bacteriocyte-like cells have been retained (Lilienstern, 1932; Jackson et al., 2021; Fukumori et al., 2022). Moreover, when endosymbionts are experimentally removed from *A. pisum* aphids and *C. floridanus* ants the bacteriocytes are still specified and migrate to the midgut epithelium (Braendle et al., 2003; Stoll et al., 2010; Rafiqi et al., 2020). Therefore, the posterior cells of the early blastoderm stage embryo may have the developmental potential due to their close proximity to both the germline (to ensure vertical transmission) and the midgut primordium (for nutritional complementation). These cells act as precursors to the bacteriocytes in multiple insects. A potentially valuable approach to address these complexities could be studies of cell lineage tracing with a focus on bacteriocytes in these insects.

Apart from the morphological evidence, the hypothetical bacteriocyte precursors appear to express conserved molecular genetic features across distantly related insects. The first line of molecular evidence comes from the gene *oskar.* This RNA binding gene is important for germline development, and is involved in the transport of mRNAs and proteins towards the formation of the germplasm in the oocyte (Ephrussi and Lehmann, 1992), but has also been shown to transport mitochondria to the posterior of the oocytes (Hurd et al., 2016). *Blochmannia* endosymbionts in *C. floridanus,* are transported to the posterior of the oocyte similarly to mitochondria and later to bacteriocytes correlating with the activity of the gene *oskar* (Rafiqi et al., 2020). The second genetic line of evidence comes from the involvement of highly conserved Hox genes in the formation of bacteriocytes in aphids, stink bugs as well as carpenter ants (Braendle et al., 2003; Matsuura et al., 2015; Rafiqi et al., 2020). The fact that such diverged lineages involve homologous genes for the specification of bacteriocytes indicates that the cell type involved may be homologous as well. We propose that this is a case of exaptation followed by co-option of the cell lineage, as conceptualized by (Gould and Lewontin, 1979). In this scenario, we propose that three cell types are found at the posterior syncytium namely i) germline destined pole cells (gdp), ii) midgut primordium (mp), and iii) midgut destined pole cells (mdp). Accordingly, in lineages with bacteriocytes the two latter cell types (mp and mdp) are co-opted to various degrees for making bacteriocytes (Figure 1). In conclusion, we think that the bacteriocyte as a novel cell fate appears to have originated through co-option of an ancestral cell type, which had proximity to the germline as well as the midgut primordium. Future studies in insects will allow us to dissect the molecular genetic mechanisms of the acquisition of novel bacteriocyte cell fates and their connection to the ecology and evolution of endosymbiosis. Eukaryotic and multicellular life evolved in a world where bacteria already existed and understanding how these organisms merged into one will help us understand life on earth better.
